# Meniscus Sign in Patients with Anterior Circulation Large Vessel Occlusion Stroke does not Predict Outcome

**DOI:** 10.1007/s00062-022-01183-w

**Published:** 2022-06-24

**Authors:** André Miranda, Ramy Abdelnaby, André Araújo, Marta Rodrigues, Valeria Battistella, José Mário Roriz, Carmélia Rodrigues, Martin Wiesmann, Jörg B. Schulz, Omid Nikoubashman, Arno Reich, Manuel Ribeiro, João Pinho

**Affiliations:** 1 Interventional Neuroradiology Unit, Centro Hospitalar Vila Nova de Gaia/Espinho, Vila Nova de Gaia, Portugal; 2 Department of Neurology, University Hospital, RWTH Aachen University, Pauwelsstr. 30, 52074 Aachen, Germany; 3 Stroke Unit, Centro Hospitalar Vila Nova de Gaia/Espinho, Vila Nova de Gaia, Portugal; 4 Stroke Unit, Centro Hospitalar Entre Douro e Vouga, Santa Maria da Feira, Portugal; 5 Stroke Unit, Unidade Local de Saúde do Alto Minho, Viana do Castelo, Portugal; 6 Department of Diagnostic and Interventional Neuroradiology, University Hospital, RWTH Aachen University, Aachen, Germany; 7 JARA-BRAIN Institute Molecular Neuroscience and Neuroimaging, Forschungszentrum Jülich GmbH and RWTH Aachen University, Aachen, Germany

**Keywords:** Ischemic stroke, Thrombectomy, Endovascular, Digital subtraction angiography, Prognosis

## Abstract

**Purpose:**

The angiographic appearance of the occlusion site was suggested to influence outcomes of stroke patients with large vessel occlusion (LVO) who undergo endovascular treatment (EVT). We aimed to study the impact of the meniscus sign (MS) on outcomes of stroke patients with anterior circulation LVO.

**Methods:**

Based on two prospective registries of acute ischemic stroke, we selected patients with carotid‑T, M1 or M2 occlusion who underwent EVT. Clinical characteristics and outcomes were collected from the registries or from individual records. Two independent observers blinded to outcomes assessed the presence of MS in digital subtraction angiography before thrombectomy. Angiographic and clinical outcomes of patients with and without MS were compared.

**Results:**

We included 903 patients, with median age of 78 years, 59.8% were male, median baseline NIHSS was 14 and 39.5% received intravenous thrombolysis. Patients with MS (*n* = 170, 18.8%) were more frequently female, presented with higher NIHSS scores and more frequently underwent intravenous thrombolysis. Presence of MS was significantly associated with cardioembolic etiology. Successful reperfusion, number of passes, first pass effect, procedural time, symptomatic intracerebral hemorrhage, in-hospital mortality and favorable 3‑month functional outcome were similar in the groups of patients with and without MS. In the multivariable analyses, MS was not associated with successful reperfusion (odds ratio, OR = 1.08, 95% confidence interval, CI = 0.76–1.55), first pass effect (OR = 0.96, 95%CI = 0.48–1.92) or favorable 3‑month outcome (OR = 1.40, 95%CI = 0.88–2.24).

**Conclusion:**

The presence of MS in acute ischemic stroke patients with anterior circulation large vessel occlusion who undergo EVT does not appear to influence angiographic or clinical outcomes.

**Supplementary Information:**

The online version of this article (10.1007/s00062-022-01183-w) contains supplementary material, which is available to authorized users.

## Introduction

Advances in the techniques and devices used in endovascular treatment (EVT) of acute ischemic stroke patients with large vessel occlusion have led to significant improvements in angiographic and clinical outcomes in the last 20 years [1]. In addition to technical aspects of EVT, there are also several patient-related factors that appear to influence the technical success of EVT and, consequently, may influence clinical outcomes. Factors such as vessel anatomy with increased curvature [2], increased clot burden score [3], thrombus imperviousness [4] and non-cardioembolic stroke etiology [5] have been associated with decreased reperfusion rates of EVT and/or poorer functional outcome. There are also some studies which suggest that the angiographic appearance of the occlusion site may also influence the reperfusion rates and clinical outcomes [6]. The meniscus sign is an angiographic sign with a concave appearance of the distal contrast column in the occlusion site, which has been associated not only with better reperfusion rates but also with fewer passes, shorter procedure time and better functional outcome [7, 8]; however, the significance of the meniscus sign in this setting remains unclear, because the few studies analyzing the meniscus sign were monocentric studies with small population sizes.

Our aim was to analyze the impact of meniscus sign in angiographic and clinical outcomes in patients with acute ischemic stroke with anterior circulation large vessel occlusion submitted to EVT.

## Methods

We conducted a multicentric retrospective case-control study based on prospective local registries of consecutive acute ischemic stroke patients submitted to EVT in two high volume comprehensive stroke centers during a 3-year period (July 2018–June 2021). Management protocols of acute ischemic stroke patients in both centers follow national and international recommendations [9]. Both centers use mainly a stent-retriever technique with femoral artery access as the technique of choice for the treatment of patients with large vessel occlusion. The combined stent-retriever-assisted vacuum-locked extraction technique was routinely used in one center. Only a residual proportion of patients (< 5%) are treated using the contact aspiration technique as the method of first choice. Balloon-guided catheters with proximal flow control are routinely used in both centers. In both centers, however, the choice of the technique is to the discretion of the interventionalist according to the anatomic and clinical characteristics of individual patients. Inclusion criteria were: 1) adult patients admitted for acute ischemic stroke; 2) presence of large vessel occlusion of the anterior circulation (carotid T, M1, M2) and 3) patients submitted to EVT. Exclusion criteria were: 1) occlusion of the cervical internal carotid artery; 2) tandem occlusion; 3) EVT other than mechanical thrombectomy, namely isolated intra-arterial thrombolysis or isolated stenting; 4) unavailability of the digital subtraction angiography (DSA) images for review. Baseline demographic variables, clinical variables, treatment with intravenous thrombolysis, details and time metrics of EVT and 3‑month outcome were collected from the prospective registries or directly from individual patient records when the information was not available in the registry. Information on ethnicity and cultural background was not available for collection, but the included patients stem from populations where west Europeans represent the largest ethnic and cultural group. Successful reperfusion was defined as a revised thrombolysis in cerebral infarction (TICI) of 2b, 2c or 3, and complete reperfusion was defined as TICI 3 [10]. First pass effect was defined as achievement of TICI 2c or 3 after the first thrombectomy maneuver. Procedural time was defined as the time between the arterial puncture and achievement of the best reperfusion, or the end of the procedure in case of persistent occlusion. Symptomatic intracerebral hemorrhage (sICH) was defined as an ICH occurring within 48 h after EVT and associated with an increase of ≥ 4 points in the National Institutes of Health Stroke Scale (NIHSS). Stroke etiology was defined according to the criteria used in the ASCOD classification [11] and published criteria for embolic stroke of undetermined source (ESUS) [12]. Favorable 3‑month outcome was defined as a modified Rankin scale (mRS) of 0–2 and collected from the prospective registries. Assessment of 3‑month functional outcome is carried out routinely in the outpatient clinic or through telephone interview in both centers.

### Assessment of Angiographic Clot Occlusion Signs

**Fig. 1:**
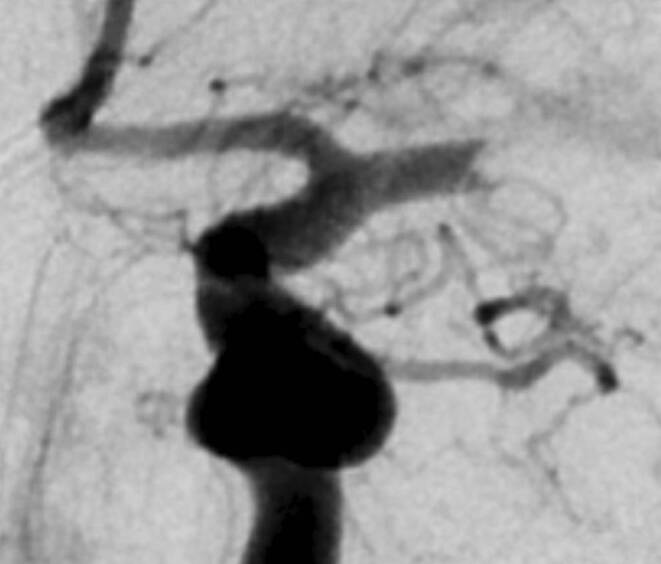
Example of the angiographic meniscus sign in a patient with an M1-occlusion

Diagnostic DSA image series before any mechanical thrombectomy maneuver were reviewed by two independent experienced observers in each center, who were blinded to patient characteristics, angiographic and clinical outcomes. Both anteroposterior and mediolateral image perspectives were systematically analyzed. The presence of meniscus sign was defined as the presence of a concave appearance of the distal contrast column in the occlusion site (Fig. 1). The tram-track sign (Supplementary Fig. 1) was also assessed because it has been considered as equivalent to the meniscus sign in some studies [7, 8]. Tram-track sign was defined as one or two-wall elongated opacification of the vessel distal to the central contrast column. Disagreements between observers were settled by consensus after joint review of the DSA images.

### Statistical Analyses

Baseline characteristics of patients with and without meniscus sign were compared using χ^2^-tests and Mann-Whitney U-tests as appropriate. Distribution of continuous variables was analyzed using the Shapiro-Wilk test. The predictive role of meniscus sign for successful reperfusion, first pass effect and favorable 3‑month outcome was examined using univariable and single-step multivariable logistic regression analyses, odds ratios (OR) and corresponding 95% confidence intervals (95% CI) were calculated. Variables thought a priori to possibly influence angiographic outcomes were included as covariates in the multivariable logistic regression model (age, sex, baseline NIHSS, intravenous thrombolysis, cardioembolic etiology). Variables thought a priori to influence clinical outcomes were included as covariates in the multivariable logistic regression model (age, sex, baseline NIHSS, baseline Alberta Stroke Program Early Computed Tomography Score, occlusion of carotid‑T, intravenous thrombolysis, sICH, cardioembolic etiology). Sensitivity analyses using the presence of meniscus and tram-track signs were also performed. Post hoc diagnostic performance analysis and univariable and single-step multivariable logistic regression analyses for the association of the meniscus sign with cardioembolic etiology were carried out. The threshold for statistical significance was set at an alpha level of 0.05. All statistical analyses were conducted using STATA version 17.0 (StataCorp LLC, College Station, TX, USA).

Retrospective studies based on the local registry of patients with acute ischemic stroke were approved by the local ethics committees (approval references 335/15 and 151/21) and, because of the retrospective nature, the ethics committees waived the need for patient-signed consent. This report follows the Strengthening the Reporting of Observational Studies in Epidemiology (STROBE) guidelines [13].

This study provides class II evidence for the significance of the angiographic meniscus sign as a marker for angiographic and clinical outcomes.

### Data Availability

Anonymized data may be provided by the corresponding author upon reasonable request for research purposes and according to the recommendations of the local ethics committees.

## Results

From a total of 1499 acute ischemic stroke patients submitted to EVT during the study period, we excluded: 197 patients because of occlusion of arteries other than those defined in the inclusion criteria, 139 patients because of tandem occlusion, 93 patients because of absent diagnostic digital subtraction angiography images, 78 patients because of isolated cervical carotid artery, 49 patients because of reperfusion after thrombolysis and 40 patients because of endovascular treatment other than mechanical thrombectomy (Supplementary Fig. 2). The final study population consisted of 903 patients, with a median age of 78 years (interquartile range, IQR = 67–85), 59.8% were male, baseline NIHSS was 14 (IQR = 9–18) and 39.5% received intravenous thrombolysis. Endovascular treatment using stent-retriever was performed in the vast majority of patients (96.8%) and successful reperfusion was achieved in 92.7% of patients. Information on functional status 3 months after stroke was available for 680 patients, and favorable outcome was achieved in 42.8%. Interobserver agreement for the presence of meniscus sign was substantial (κ = 0.76).

Meniscus sign was present in 170 patients (18.8%). Table 1 describes the baseline characteristics of patients with and without meniscus sign. Patients with meniscus sign were more frequently female (67.7% versus 58.0%, *p* = 0.021), had slightly higher NIHSS (15 versus 14, *p* = 0.043) and had been treated more frequently with intravenous thrombolysis (48.8% versus 37.4%, *p* = 0.006). Patients with meniscus sign had more frequently a cardioembolic etiology for stroke (76.0% versus 65.0%, *p* = 0.008) and less frequently had intracranial stenosis at the site of occlusion (0.6% versus 6.4%, *p* = 0.003). The association of meniscus sign and cardioembolic etiology persisted after adjustment for relevant confounders (Supplementary Table 1). The meniscus sign presented a sensitivity of 21.3%, a specificity of 86.3%, a positive predictive value of 75.9% and a negative predictive value of 35.0% for the presence of cardioembolic etiology.

**Table 1:** Characteristics of the study population according to the presence of the meniscus sign

	No meniscus sign (*n* = 733)	Meniscus sign (*n* = 170)	*p*
*Male sex*	308 (42.0)	55 (32.3)	0.021
*Age (years)*	78 (67–85)	79 (71–84)	0.346
*Hypertension*	537 (73.4)	131 (77.1)	0.322
*Diabetes*	183 (25.0)	38 (22.4)	0.470
*Dyslipidemia*	281 (38.3)	59 (34.7)	0.379
*Baseline anticoagulation*	181 (24.7)	36 (21.2)	0.334
*Intravenous thrombolysis*	274 (37.4)	83 (48.8)	0.006
*Baseline NIHSS score*	14 (9–18)	15 (10–19)	0.043
*Baseline ASPECTS score*	9 (8–10)	9 (7–10)	0.477
*Large vessel occlusion site*	–	–	0.307
Carotid T	109 (14.9)	33 (19.4)	–
M1	387 (52.8)	82 (48.2)	–
M2	237 (32.3)	55 (32.4)	–
*Onset-to-groin puncture time (minutes)*	224 (150–405)	234 (146–372)	0.964
*Ischemic stroke etiology* ^a^	–	–	–
Cardioembolic	444 (65.0)	120 (76.0)	0.008
Cervical large vessel disease	31 (4.5)	5 (3.2)	0.442
Intracranial stenosis	44 (6.4)	1 (0.6)	0.003
Dissection	7 (1.0)	0	0.201
ESUS	135 (19.8)	30 (19.0)	0.824
Other cause	22 (3.2)	2 (1.3)	0.183

Values are presented as *n* (%) or median (interquartile range)

*NIHSS* National Institutes of Health Stroke Scale, *ESUS* embolic stroke of undetermined source, *ASPECTS* Alberta Stroke Program Early Computed Tomography Score

^a^ Missing etiology in 62 patients because of incomplete investigation

The angiographic outcomes between the two groups did not differ significantly (Table 2). Achievement of successful reperfusion, complete reperfusion, number of passes, and first pass effect were similar in patients with and without meniscus sign. There was a nonsignificant trend for slightly faster procedural times in patients with meniscus sign (median time 33 min vs. 40 min, *p* = 0.092). The site of the occlusion did not influence successful reperfusion, complete reperfusion, first pass effect, number of passes or procedural time in patients with and without meniscus sign (Supplementary Table 2). Likewise, we found no difference in periprocedural complications, in the occurrence of sICH or in-hospital mortality. The 3‑month favorable outcome was similar in both groups (Table 2).

**Table 2:** Angiographic and clinical outcomes according to the presence of the meniscus sign

	No meniscus sign (*n* = 733)	Meniscus sign (*n* = 170)	*p*
**Angiographic outcomes**			
*Number of passes*	1 (1–2)	1 (1–2)	0.645
*Number of passes ≥* *3*	171 (23.3)	35 (20.6)	0.443
*First pass effect*	338 (46.1)	85 (50.0)	0.360
*Successful reperfusion*	679 (92.6)	158 (92.9)	0.889
*Complete reperfusion*	462 (63.0)	110 (64.7)	0.683
*Procedural time (min)*	40 (24–66)	33 (23–55)	0.092
*Periprocedural complications*	–	–	0.165
Arterial perforation	12 (1.7)	2 (1.2)	–
Embolization to different territory	5 (0.7)	4 (2.4)	–
Dissection	22 (3.0)	8 (4.7)	–
**Clinical outcomes**			
*Symptomatic intracerebral hemorrhage*	43 (5.9)	12 (7.1)	0.558
*In-hospital mortality*	110 (15.0)	22 (12.9)	0.492
*Favorable 3‑month outcome* ^a^	228 (41.5)	63 (48.1)	0.173

Values are presented as *n* (%) or median (interquartile range)

^a^ 3-month functional status available for 680 patients

In the univariable logistic regression analysis presence of meniscus sign was not associated with first pass effect, with successful reperfusion or with favorable 3‑month outcome. Presence of meniscus sign was also not associated with first pass effect, successful reperfusion or favorable 3‑month outcome in the multivariable logistics regression analyses (Table 3). When patients with meniscus sign and with tram-track sign were analyzed as one group, the angiographic and clinical outcome results did not change significantly comparing to the analyses of patients with meniscus sign only (Supplementary Tables 3 and 4).

**Table 3:** Univariable and multivariable logistic regression analyses for meniscus sign as a predictor of successful reperfusion and first pass effect

	Odds ratio (95% confidence interval)	*p*
*Successful reperfusion*		
Meniscus sign (unadjusted)	1.17 (0.84–1.63)	0.360
Meniscus sign (adjusted) ^a^	1.08 (0.76–1.55)	0.657
*First pass effect*		
Meniscus sign (unadjusted)	1.04 (0.55–2.00)	0.889
Meniscus sign (adjusted) ^a^	0.96 (0.48–1.92)	0.898
*Favorable 3‑month outcome*		
Meniscus sign (unadjusted)	1.30 (0.89–1.91)	0.173
Meniscus sign (adjusted) ^b^	1.40 (0.88–2.24)	0.159

^a^ Adjustment for age, sex, baseline National Institutes of Health Stroke Scale, intravenous thrombolysis, cardioembolic etiology

^b^ Adjustment for age, sex, baseline National Institutes of Health Stroke Scale, baseline Alberta Stroke Program Early Computed Tomography Score, occlusion of carotid‑T, intravenous thrombolysis, symptomatic intracerebral hemorrhage, cardioembolic etiology.

## Discussion

The main conclusion of our study is that the meniscus sign does not influence angiographic outcomes and is not a predictor of clinical outcomes in acute ischemic stroke patients with anterior circulation large vessel occlusion who receive EVT. The presence of meniscus sign in DSA before mechanical thrombectomy was not associated with achievement of successful reperfusion, complete reperfusion first pass effect or number of passages, and we only found a nonsignificant trend for slightly longer procedural times in these patients. The presence of the meniscus sign was also not associated with angiographic outcomes when different arterial occlusion sites (carotid T, M1, M2) were individually analyzed. In addition, no association of meniscus sign and periprocedural complications, sICH, intrahospital mortality or favorable 3‑month functional outcome was found. Our results confirm a high angiographic efficacy of EVT in a real-world setting, which is probably a result of a continuous improvement of techniques and devices and may render irrelevant the angiographic appearance of occlusion site.

The current limited available evidence suggested, however, an association between the presence of the meniscus sign and better outcomes. In a cohort of 89 patients with basilar artery occlusion, the meniscus sign, which occurred more frequently in distal occlusions, was found to be an independent predictor of successful reperfusion, and was associated with shorter procedural times, higher incidence of first pass effect and lower number of passes [7]. Specific characteristics of the basilar artery occlusions may mediate these findings, namely higher frequency of underlying large vessel disease and need for intracranial stenting [14]. We found that the meniscus sign was rare in the presence of underlying intracranial stenosis and is significantly associated with cardioembolic etiology, which is a known independent predictor of successful reperfusion [5]. In cohorts where the proportion of intracranial atherosclerotic stenosis is higher, such as in Asian stroke patients [15], a significant proportion of patients with large vessel occlusion and no meniscus sign will have an in situ thrombosis in an intracranial atherosclerotic plaque and/or stenosis, which may be associated with reduced efficacy of EVT [16]. Likewise, lower reperfusion rates are expected to be achieved in patients with basilar artery occlusion and no meniscus sign, because a high proportion of these patients will present a severe basilar artery stenosis, which is known to be responsible for 1/4 of basilar artery occlusion cases [17]. We emphasize that our results are, therefore, mainly valid for occlusions of the anterior circulation and for west European populations.

Another study which analyzed the angiographic appearance of the occlusion site in patients with M1 or M2 occlusion who received EVT showed that the presence of the meniscus and the tram-track signs was associated with a lower number of passes and with better 3‑month functional outcome [8]. This study is, however, limited because of the small size of the study population (144 patients included of whom only 18 presented meniscus or tram-track sign).

Other smaller studies also examined the significance of angiographic occlusive signs in patients with large vessel occlusion who underwent EVT; however, a specific evaluation of the meniscus sign was not performed [18, 19]. Although a higher prevalence of the meniscus sign was reported in patients with basilar artery occlusion (63%) [7], the prevalence of 19% we found in the anterior circulation is more in line with the prevalence of 13% and 34% found by Mönch et al. [8] and Garcia-Bermejo et al. [19], respectively. These three studies had significantly smaller study populations in comparison to our study, but the prevalence of meniscus sign will probably vary according to the territory of the occlusion and to the relative distribution of different stroke etiologies in each study.

In an older study of 33 patients treated with intra-arterial thrombolysis, the presence of meniscus sign (found in 6 patients) required lower doses of urokinase for achievement of reperfusion and was associated with higher rates of at least partial reperfusion [20]. The reason for patients who received intravenous thrombolysis to have more frequently meniscus sign in our study remains unclear. We excluded a possible effect of time between symptom onset and groin puncture in the presence of meniscus sign. If it could represent a sign for a higher thrombus susceptibility to thrombolysis remains speculative. A possible link supporting this hypothesis is that thrombi with cardioembolic origin, usually rich in fibrin and platelets, also have an increased perviousness in computed tomography [21], which is known to be associated with higher success of intravenous thrombolysis [22]. Although, in our study, the meniscus sign was more frequent in patients with cardioembolic stroke etiology, studies report conflicting results in what the association between meniscus sign and thrombus perviousness is concerned [8, 23].

The independent association of the meniscus sign with cardioembolic etiology was not previously reported in the literature, even though Garcia-Bermejo et al. described a higher frequency of cardioembolic etiology in patients with non-tapered occlusions (which include the meniscus sign) [19]. Even though specificity was relatively high, the diagnostic performance of the meniscus sign for the presence of underlying cardioembolic etiology was not optimal; however, the aim of our study was not to specifically address biomarkers for cardioembolism. The likelihood of an underlying intracranial stenosis appears to be low in the presence of the meniscus sign, unlike the taper sign (beak-like or flame-like occlusion appearance), which has been associated with intracranial atherosclerotic lesions and dissections [6]. These associations between the angiographic appearance of the occlusion site and stroke etiology must be interpreted with caution and confirmed in independent cohorts with adjustment for other important variables which were not evaluated in our study.

The reasons for the associations we found between meniscus sign and female sex and higher NIHSS scores remain unclear but we speculate it could be mediated by differential distribution of stroke etiology or by chance.

It has been suggested that the angiographic occlusion appearance could influence the choice of EVT technique. Baik et al. found that in 161 patients with basilar artery occlusion, the use of contact aspiration as the primary thrombectomy technique in patients with meniscus sign was associated with shorter procedural time, lower number of maneuvers, higher frequency of first pass effect and higher frequency of complete reperfusion, when compared to the use of stent-retrievers [24]. Successful reperfusion was, however, not different between the two different primary techniques and clinical outcomes were similar. A recent study of patients with M1 and M2 occlusions showed better complete recanalization rates and better functional outcomes in patients with meniscus sign who had been treated with contact aspiration in comparison to stent-retriever [23]. Although contact aspiration technique in patients with meniscus sign was independently associated with complete recanalization, the analysis for the prediction of outcome was not adjusted for relevant co-variates in this study. The validity of these study results is also limited by the low number of patients with meniscus sign included (26 in 111 patients who underwent thrombectomy), and low number of patients with meniscus sign treated with each technique (14 with contact aspiration and 12 with stent-retriever). In our study we could not test the benefit of using stent-retriever versus contact aspiration in patients with meniscus sign, because only a residual number of patients were treated with the latter technique. It remains an open discussion if contact aspiration could provide better angiographic and clinical outcomes in patients with meniscus sign.

The main limitations of our study include the fact that we did not analyze other potential factors which are known to influence the success of EVT, namely anatomy and curvature of the occluded vessels, the clot burden and thrombus perviousness; however, because the meniscus sign was not associated with angiographic outcomes or clinical outcomes, it is unlikely that the adjustment of our analyses for these additional parameters would change our results significantly. The fact that the use of stent-retrievers was the primary technique of choice for the vast majority of patients in both centers did not allow to study the possible differential benefit of contact aspiration in patients with meniscus sign. Other limitations include missing information related to 3‑month outcome in 1/4 of the patients and incomplete etiological investigation in 7% of the patients. Our study also has significant strengths, namely the fact that it is based on prospective registries of stroke patients, that it is multicentric, that the population size is relatively large, and that assessment of the meniscus sign was blinded for angiographic and clinical outcomes.

## Conclusion

The presence of meniscus sign in acute ischemic stroke patients with anterior circulation large vessel occlusion who undergo EVT with a stent-retriever technique does not appear to influence angiographic outcomes such as successful reperfusion and first pass effect, or clinical outcomes such as sICH and 3‑month functional outcome. The meniscus sign appears to be independently associated with cardioembolism, and this association warrants further studies.

## Supplementary Information

Supplementary Table 1: Univariable and multivariable logistic regression analyses for meniscus sign as a predictor of cardioembolic etiology, Supplementary Table 2: Angiographic outcomes according the presence of meniscus sign and occlusion site, Supplementary Table 3: Angiographic and clinical outcomes according to the presence of meniscus or tram-track sign, Supplementary Table 4: Univariable and multivariable logistic regression analyses for meniscus or tram-track sign as a predictor of first pass effect and successful reperfusion, Supplementary Figure 1: Example of the angiographic tram-track sign in a patient with an intracranial carotid occlusion, Supplementary Figure 2: Patient flow diagram
