# Supplementary material for: Meniscus Sign in Patients with Anterior Circulation Large Vessel Occlusion Stroke does not Predict Outcome
Source: Clin Neuroradiol. 2022 Jun 24;33(1):65–72. doi: 10.1007/s00062-022-01183-w (PMC10014662; doi:10.1007/s00062-022-01183-w)

**SUPPLEMENTARY MATERIAL**

**Supplementary Table 1:** Univariable and multivariable logistic regression analyses for meniscus sign as a predictor of cardioembolic etiology

|  | **Odds ratio**  **(95% confidence interval)** | **p** |
| --- | --- | --- |
| Cardioembolic etiology |  |  |
| Meniscus sign (unadjusted) | 1.70 (1.14-2.53) | 0.009 |
| Meniscus sign (adjusted)^†^ | 1.81 (1.16-2.82) | 0.009 |

^†^ Adjustment for age, sex, hypertension, baseline National Institutes of Health Stroke Scale, baseline anticoagulation, intravenous thrombolysis

**Supplementary Table 2:** Angiographic outcomes according the presence of meniscus sign and occlusion site

|  | **No meniscus sign** | **Meniscus sign** | **p** |
| --- | --- | --- | --- |
| Successful reperfusion |  |  |  |
| Carotid T | 100 (91.7) | 27 (81.8) | 0.104 |
| M1 | 360 (93.0) | 78 (95.1) | 0.487 |
| M2 | 219 (92.4) | 53 (96.4) | 0.295 |
| Complete reperfusion |  |  |  |
| Carotid T | 70 (64.2) | 15 (45.5) | 0.054 |
| M1 | 268 (69.3) | 62 (75.6) | 0.252 |
| M2 | 124 (52.3) | 33 (60.0) | 0.303 |
| First pass effect |  |  |  |
| Carotid T | 44 (40.4) | 10 (30.3) | 0.297 |
| M1 | 190 (49.1) | 44 (53.7) | 0.453 |
| M2 | 104 (43.9) | 31 (56.4) | 0.094 |
| Number of passes |  |  |  |
| Carotid T | 1 (1-3) | 2 (1-3) | 0.202 |
| M1 | 1 (1-2) | 1 (1-2) | 0.686 |
| M2 | 1 (1-2) | 1 (1-2) | 0.142 |
| Procedural time |  |  |  |
| Carotid T | 42 (25-80) | 38 (21-81) | 0.677 |
| M1 | 37 (23-66) | 31 (23-50) | 0.686 |
| M2 | 41 (27-60) | 35 (25-49) | 0.142 |

**Supplementary Table 3**: Angiographic and clinical outcomes according to the presence of meniscus or tram-track sign

|  | **No meniscus / tram-track sign (n=613)** | **Meniscus / tram-track sign**  **(n=290)** | **p** |
| --- | --- | --- | --- |
| *Angiographic outcomes* |  |  |  |
| Number of passes | 1 (1-2) | 1 (1-2) | 0.188 |
| Number of passes ≥3 | 142 (23.2) | 64 (22.1) | 0.714 |
| First pass effect | 281 (45.8) | 142 (49.0) | 0.380 |
| Successful reperfusion | 567 (92.5) | 270 (93.1) | 0.743 |
| Complete reperfusion | 390 (63.6) | 182 (62.8) | 0.802 |
| Procedural time | 40 (24-66) | 36 (24-57) | 0.213 |
| Periprocedural complications |  |  | 0.067 |
| Arterial perforation | 10 (1.7) | 4 (1.4) |  |
| Embolization to different territory | 4 (0.7) | 5 (1.7) |  |
| Dissection | 14 (2.3) | 16 (5.6) |  |
| *Clinical outcomes* |  |  |  |
| Symptomatic intracerebral hemorrhage | 34 (5.5) | 21 (7.2) | 0.320 |
| In-hospital mortality | 96 (15.7) | 36 (12.4) | 0.197 |
| Favourable 3-month outcome | 188 (41.1) | 103 (46.2) | 0.211 |

Values are presented as n (%) or median (interquartile range)

**Supplementary Table 4**: Univariable and multivariable logistic regression analyses for meniscus or tram-track sign as a predictor of first pass effect and successful reperfusion

|  | **Odds ratio**  **(95% confidence interval)** | **p** |
| --- | --- | --- |
| Successful reperfusion |  |  |
| Meniscus sign / tram-track sign (unadjusted) | 1.10 (0.64-1.89) | 0.743 |
| Meniscus sign / tram-track sign (adjusted)^*^ | 1.07 (0.59-1.91) | 0.829 |
| First pass effect |  |  |
| Meniscus sign / tram-track sign (unadjusted) | 1.13 (0.86-1.50) | 0.380 |
| Meniscus sign / tram-track sign (adjusted)^*^ | 1.03 (0.76-1.38) | 0.861 |
| Favourable 3-month outcome |  |  |
| Meniscus sign / tram-track sign (unadjusted) | 1.23 (0.89-1.70) | 0.212 |
| Meniscus sign / tram-track sign (adjusted)^†^ | 1.14 (0.78-1.68) | 0.497 |

^*^ Adjustment for age, sex, baseline National Institutes of Health Stroke Scale, intravenous thrombolysis, cardioembolic etiology

^†^ Adjustment for age, sex, baseline National Institutes of Health Stroke Scale, baseline Alberta Stroke Program Early Computed Tomography Score, occlusion of carotid-T, intravenous thrombolysis, symptomatic intracerebral hemorrhage, cardioembolic etiology.

**Supplementary Figure 1**: Example of the angiographic tram-track sign in a patient with an intracranial carotid occlusion


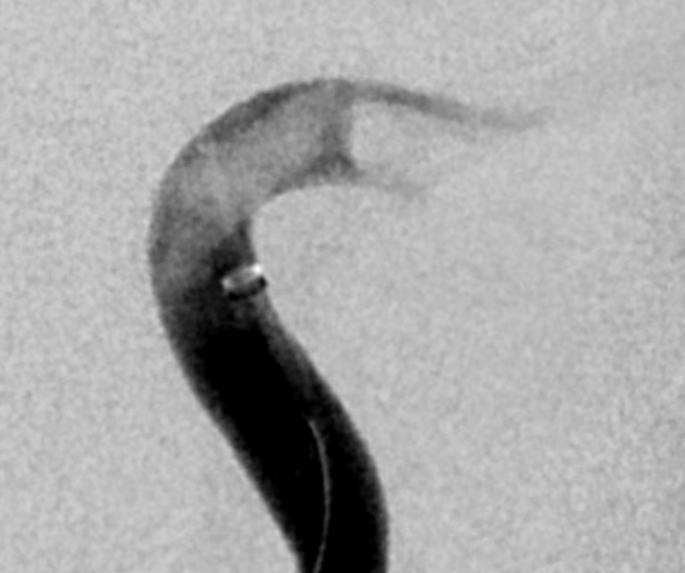


**Supplementary Figure 2**: Patient flow diagram


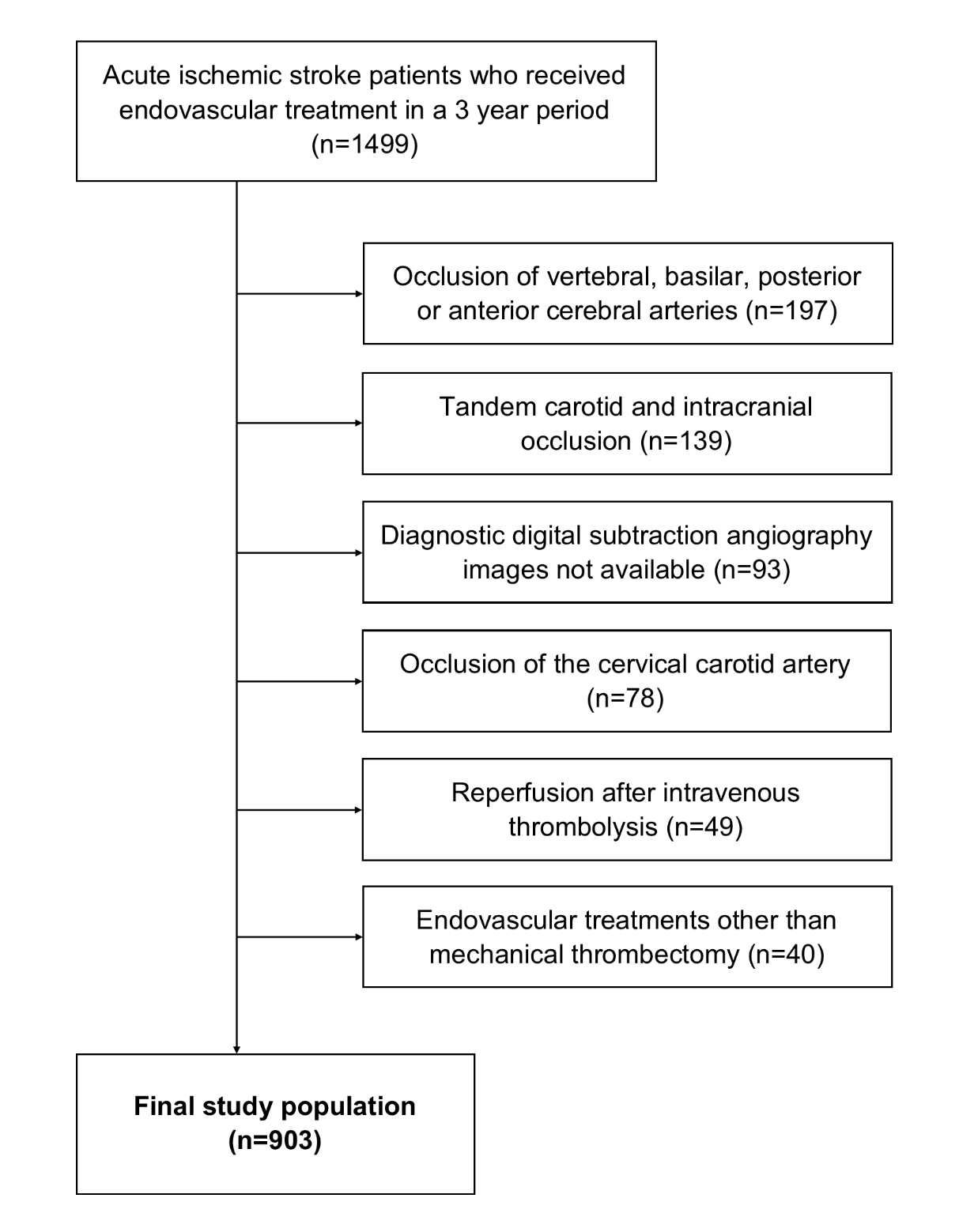

Supplement: Supplementary file 1 — Supplementary Table 1: Univariable and multivariable logistic regression analyses for meniscus sign as a predictor of cardioembolic etiology, Supplementary Table 2: Angiographic outcomes according the presence of meniscus sign and occlusion site, Supplementary Table 3: Angiographic and clinical outcomes according to the presence of meniscus or tram-track sign, Supplementary Table 4: Univariable and multivariable logistic regression analyses for meniscus or tram-track sign as a predictor of first pass effect and successful reperfusion, Supplementary Figure 1: Example of the angiographic tram-track sign in a patient with an intracranial carotid occlusion, Supplementary Figure 2: Patient flow diagram [file 62_2022_1183_MOESM1_ESM.docx]
